# E-cigarettes, a safer alternative for teenagers? A UK focus group study of teenagers' views

**DOI:** 10.1136/bmjopen-2016-013271

**Published:** 2016-11-16

**Authors:** Shona Hilton, Heide Weishaar, Helen Sweeting, Filippo Trevisan, Srinivasa Vittal Katikireddi

**Affiliations:** 1 MRC/CSO Social and Public Health Sciences Unit, University of Glasgow, Scotland, UK; 2 American University, School of Communication, Washington, District of Columbia, USA

**Keywords:** E-cigarettes, vaping, teenagers, understandings, smoking

## Abstract

**Objective:**

Concerns exist that e-cigarettes may be a gateway to traditional cigarettes and/or (re)normalise teenage smoking. This qualitative study explores how teenagers in the UK currently perceive e-cigarettes and how and why they do or do not use them.

**Design:**

16 focus groups were conducted across the UK between November 2014 and February 2015, with 83 teenagers aged 14–17. All discussions were digitally recorded, transcribed verbatim, imported into NVivo 10 and thematically analysed.

**Results:**

Teenagers generally agreed that e-cigarettes are useful products for smokers, including teenage smokers, to quit or reduce traditional cigarette use. Concerns were expressed about lack of information on their precise ingredients and any unknown risks for users and bystanders. However, teenagers typically viewed e-cigarettes as substantially less harmful than traditional cigarettes. They perceived e-cigarettes as attractive, with products described as ‘fun’ and having ‘great flavourings’. Seeing websites or social media featuring e-cigarettes, especially YouTube ‘vaping tricks’, prompted some experimentation and imitation. E-cigarettes were used in a variety of situations, including at parties or when they could not smoke traditional cigarettes. A very few participants suggested covert use was a possibility and that e-cigarettes might help maintain a fledgling nicotine habit.

**Conclusions:**

Teenagers support the use of e-cigarettes as smoking cessation aids for established adult smokers. However, they engage with these products differently from adults, with the novel hypothesis that covert use could potentially reinforce traditional cigarette smoking requiring further investigation. Policy responses should more clearly meet the needs of young people, as well as helping established adult smokers.

**Strengths and limitations of this study**

- Understanding how teenagers perceive and use e-cigarettes is a research priority, given the potential lifelong consequences of behaviours established at this age, but little qualitative research has been conducted.
- This study provides one of the first indepth explorations of teenagers' lived experiences and perceptions of e-cigarettes.
- In consideration of the gateway theory, it is worth noting that this sample is not representative of British youth and the sample size did not allow detailed analyses of differences moving into smoking or opinions with regard to smoking status, e-cigarette use and socioeconomic variables. To what extent the experiences and perceptions of UK youth differ from those of young people in other countries remains to be explored.

## Introduction

Electronic nicotine delivery systems (e-cigarettes) have become increasingly popular.^1–3^ Evidence on their potential benefits and harms is limited, and the views of public health researchers and advocates are divided,^4–7^ with controversial academic and public debates about e-cigarettes escalating recently. Proponents argue that e-cigarettes are 95% less harmful than tobacco,^8^ ^9^ and useful adjuncts to help smokers quit;^10–12^ opponents fear they may encourage tobacco smoking uptake and maintenance, especially among teenagers.^6^ ^13–17^

Concerns that e-cigarettes may encourage a transition into tobacco smoking and that they may (re-)normalise smoking behaviours lie at the centre of the debate about their possible harms for teenagers.^6^ ^13–17^ Fears also exist that tobacco producers may use e-cigarettes to influence public health policy and as a marketing opportunity to target young people, a group with high proclivity towards risky behaviours.^18–20^ As such, there is a growing need for indepth research on young people's perspectives on e-cigarettes to ensure responsive tobacco control policy aimed at young people. A recent systematic review of public awareness, use, reactions and beliefs about e-cigarettes found most relevant research focuses on the US context and investigates perceptions of e-cigarettes among adult tobacco smokers.^21^ Research which uses qualitative methods to explore how e-cigarettes are perceived by different population groups is particularly rare, with only a small number of studies focusing on young people's perceptions and use of e-cigarettes.^22^ Understanding how teenagers perceive and use e-cigarettes is a research priority, given most adult smokers start at this point in the life course.^23^ ^24^ This UK-based study therefore sought to qualitatively explore teenagers' perceptions of e-cigarettes and how and why they used them at a time when the regulation of e-cigarettes was high on the political agenda.

## Methods

Sixteen focus groups were conducted (November 2014 to February 2015), 11 in Scotland and 5 in England. Friendship groups of 4–7 participants were used to facilitate indepth insights and promote participant interaction. Purposive sampling was used to recruit a diverse range of teenagers in terms of socioeconomic background, gender, users/non-users of traditional cigarettes and/or e-cigarettes. To ensure the inclusion of enough participants with experiences of smoking or/and e-cigarette use, half of the groups were recruited from more socioeconomically deprived areas also associated with higher uptake of smoking. All the participants were recruited from small local community groups, with 2 groups being recruited directly by researchers and the other 14 groups being recruited through youth leaders/organisers handing out information. Participants were each given £20 vouchers to cover travel expenses and as appreciation for their time. The study and all its documentation obtained ethical approval from the University of Glasgow, College of Social Sciences. Informed written consent was obtained from every participant and one of their respective parents or guardians prior to taking part in the group discussion.

At the start of each group, participants completed a brief questionnaire which included fixed-choice questions, adapted from the Scottish Schools Adolescent Lifestyles and Substance Use Survey about their use of traditional cigarettes and e-cigarettes (‘do you smoke cigarettes/use an e-cigarette at all nowadays’, and ‘which statement describes you best’: never tried/used, not even a puff or two/not even once; once had a puff or two/once or twice tried, but never smoke/use now; do sometimes smoke/use).^25^ While such categorisations, which we describe here as ‘never’, ‘tried’ and ‘current’, may not exactly match young people's own smoking identity, they are indicative of the categories.^26^ These was complete agreement in respect of reporting current smoking and sometimes smoking and only one discrepancy in respect of reporting current e-cigarette use and sometimes using e-cigarettes (one ‘current’ user reported having tried once or twice, coded as ‘tried’).

A topic guide, based on past literature and pilot work, explored themes including: knowledge and understandings of e-cigarettes; beliefs about potential benefits and harms of e-cigarettes; and experiences of e-cigarette use. Promotional advertising materials (posters, still images from television and online adverts) were used to help stimulate discussion. All discussions were facilitated by the same experienced qualitative researcher (FT), lasted 40–70 min, were digitally recorded (with participants' permission) and transcribed verbatim. Transcripts were checked against the recordings for accuracy, and participants identified and given pseudonyms. Each transcript was imported into NVivo 10, thematically coded and cross-checked by the research team for agreement about the codes. This approach ensured a rigorous method to develop agreement on coding, discuss common reasoning and identify ideas specific to certain subgroups or individuals, or related to the literature. Using Glaser's constant comparative approach to analysis,^27^ attention was paid to any deviant/contradictory cases and to group dynamics using the transcripts supplemented by field-note observations.

## Results

Eighty-three individuals aged 14–17 years participated (44 males, 39 females), representing diversity in sociodemographic characteristics and smoking-related behaviours (table 1). It is notable that all current e-cigarette users used nicotine in their e-cigarettes and were also current smokers. Around a third of all current smokers were current dual users, all but one current smoker had experience of e-cigarettes. The targeted approach to recruitment meant that only eight never smokers in the study had ever tried an electronic cigarette (table 2). Of those participants that had used e-cigarettes, they had either purchased them over the internet or been given them from other teenagers. While all of the teenagers were well aware of vape shops, none of them mentioned having used a vape shop themselves to purchase e-cigarettes.

**Table 1:** Focus group location, participants and their cigarette smoking and e-cigarette use

Location	Pseudonym	Age	Cigarette smoker	E-cig use
Glasgow SW Scotland	James	15	Never	Never
	Gerry	14	Never	Never
	Chrissie	14	Never	Never
	Janice	15	Never	Never
Glasgow SW Scotland	Felicity	16	Never	Never
	Donald	16	Never	Never
	Mitchell	16	Never	Never
	Louis	16	Tried	Never
Glasgow SW Scotland	Thomas	16	Never	Never
	Alice	17	Tried	Tried
	Joshua	17	Never	Never
	Lachlan	16	Never	Never
	David	16	Tried	Tried
Glasgow SW Scotland	Robert	14	Never	Never
	Liam	14	Never	Tried
	Graham	14	Never	Never
	Adrian	15	Never	Never
	John	14	Tried	Never
Glasgow SW Scotland	Maria	16	Never	Tried
	Oliver	16	Never	Never
	Allan	17	Never	Never
	Clare	17	Tried	Tried
	Jamie	17	Current	Never
	Roslyn	17	Tried	Tried
Edinburgh SE Scotland	Iain	17	Current	Tried
	Michael	17	Current	Tried
	Stewart	17	Current	Current
	Richard	17	Current	Tried
	Hannah	17	Current	Tried
Edinburgh SE Scotland	Hayley	14	Current	Tried
	Lucy	14	Current	Tried
	Jennifer	14	Current	Tried
	Francis	14	Current	Tried
	Judith	14	Current	Tried
	Stuart	15	Never	Never
	Niall	14	Current	Tried
Edinburgh SE Scotland	Lorna	16	Current	Tried
	Sharon	16	Current	Tried
	Ben	16	Current	Tried
	Wendy	15	Current	Tried
	Christine	17	Current	Tried
Edinburgh SE Scotland	Libby	16	Current	Tried
	Fergus	17	Current	Current
	Isaac	17	Never	Never
	Helen	17	Tried	Never
	Matthew	17	Current	Tried
Kirkcaldy SE Scotland	Caiomhe	15	Never	Never
	Lauren	16	Never	Never
	Timothy	16	Current	Tried
	Danyul	16	Current	Current
	Lesley	17	Tried	Never
	Jonathan	17	Never	Never
Kirkcaldy SE Scotland	Fiona	16	Current	Current
	Scott	17	Current	Tried
	Murray	16	Current	Current
	Louisa	16	Current	Current
	Ella	16	Current	Current
	Aisha	14	Current	Current
	Maggie	15	Current	Current
Greater Manchester NW England	Rhyan	17	Tried	Tried
	Charity	16	Never	Never
	Elizabeth	15	Never	Never
	Katie	17	Tried	Never
	Harriet	17	Never	Never
Greater Manchester NW England	Jane	16	Never	Never
	Darnell	14	Never	Tried
	George	16	Tried	Tried
	Emma	17	Tried	Tried
	Paul	17	Never	Tried
Merseyside NW England	Finlay	15	Never	Never
	Sara	15	Never	Never
	Steven	14	Never	Never
	Katherine	14	Never	Never
Newcastle NE England	Fraser	14	Never	Tried
	Susan	15	Never	Tried
	Karen	17	Tried	Tried
	Drew	16	Never	Tried
	Alex	17	Current	Tried
Newcastle NE England	Rosie	17	Tried	Never
	Carla	14	Tried	Tried
	Mairi	15	Never	Tried
	Gregor	17	Current	Current
	Henry	14	Never	Never

**Table 2:** E-cigarette use according to cigarette smoking—numbers (column and row percentages)

	E-cigarette use											
	Never			Tried			Current			Total		
Cigarette smoker	N	(col %)	(row %)	N	(col %)	(row %)	N	(col %)	(row %)	N	(col %)	(row %)
Never	**29**	(81)	(78)	**8**	(22)	(22)	**0**	(0)	(0)	**37**	(45)	(100)
Tried	**6**	(17)	(40)	**9**	(24)	(60)	**0**	(0)	(0)	**15**	(18)	(100)
Current	**1**	(3)	(3)	**20**	(54)	(65)	**10**	(100)	(32)	**31**	(37)	(100)
Total	**36**	(100)	(43)	**37**	(100)	(45)	**10**	(100)	(12)	**83**	(100)	(100)

Participants appeared highly engaged and eager to discuss e-cigarettes. When asked what influenced their opinions, the media were commonly cited, with social media sources such as Facebook, Twitter and YouTube specifically mentioned, alongside the internet and stories from families and friends. In many discussions, teenagers drew on first-hand experiences and observations of their social circles. There was general agreement that e-cigarettes were a beneficial product for long-term smokers who wanted to quit or cut down smoking. However, most of the discussions focused on their potential harms, with a particular emphasis on young people and children. Participants frequently questioned, challenged and amended each other's statements, reflecting high levels of openness and ambiguity about e-cigarettes.

This paper presents, in turn, the four most dominant themes: (1) perceptions of the potential harms of e-cigarettes; (2) appeal of e-cigarettes to children and teenagers; (3) acknowledgement of e-cigarette experimentation, use and dual use among different user groups; (4) interplay between online and real-world experiences.

### Perceptions of the potential harms of e-cigarettes

Potential health harms and unknown harmful ingredients were a common theme, spontaneously expressed in 12 focus groups. Conversations focused on the lack of clarity over e-cigarette composition and potential risks to users and bystanders, with an acknowledgement that this uncertainty arose from their relative novelty. In five groups, participants hypothesised that future research might show e-cigarettes to be more harmful than currently acknowledged. In one instance, this claim was underpinned by a direct comparison with changing evidence on tobacco and the resulting increasing public awareness of harm:

Robert “When original cigarettes came out they didnae know it was bad for you, they were actually encouraging you tae smoke.” (14, never-smoker, never e-cigarettes)

Liam “An’ then so the same point is, and obviously everybody knows here that smoking's bad, like normal cigarettes.” (14, never-smoker, tried e-cigarettes)

Robert “And you're saying now when these things [e-cigs] came out how dae they know these arenae gonnae be the same thing?”

Uncertainty was reflected in responses to questions and prompts about the health harms of e-cigarettes, with young people frequently framing these as questions, to the facilitator and each other, as the following exchange about e-cigarette content illustrates:

Finlay “They are just like a normal cigarette, really, isn't it? It's just electric. Like, you can put whatever you want in there and smoke it. It's like, people put all sorts in there, really? […]” (15, never-smoker, never e-cigarettes)

Sara “Doesn't it just like vaporise what's in a normal cigarette?” (15, never-smoker, never e-cigarettes)

There was some discussion about whether e-cigarettes might be more or less addictive than traditional cigarettes and it was common for participants to acknowledge that they were unsure but that they would assume them to be less addictive than traditional cigarettes. Across the groups, participants offered a range of possible ingredients and chemicals that they thought e-cigarettes might contain, including: ‘oils’, ‘flavourings’, ‘water’, ‘nicotine’, ‘sugar’, ‘liquid anti-freeze’, ‘tar’, ‘carbon monoxide’, ‘alcohol’, ‘tobacco’ and other ‘bad things’*.* Ingredients were described as producing potentially harmful ‘toxins’, ‘poisons’ and ‘poisonous gases’. Despite uncertainties about the exact harms of e-cigarettes, there was general consensus that traditional cigarettes were more harmful than vaping, with vapour being “obviously not as harmful as the smoke from [a] normal cigarette” (Gerry, 14, never-smoker, never e-cigarettes).

Uncertainty and acknowledgement of lack of evidence about the harmful effects of e-cigarettes also extended to the potential effects of vapour on bystanders. Murray (16, current smoker, current e-cigarettes) mentioned: “we need to find out a bit more about, like, the chemicals that are in it to, like, know whether you'd want to be around vapers.” In two groups, participants expressed concern about potential harmful chemical reactions and unknown dangers of secondhand vapour exposure.

I don't really like the idea of using e-cigarettes, smoking around me, because you don't really know what the sort of vapour that they exhale can do to you. (Alice, 17, tried-smoking, tried e-cigarettes)

[…] I think it is still unknown so I don't think it's good for people to smoke [vape] these e-cigarettes around like other people who will inhale it. (Joshua, 17, never-smoker, never e-cigarettes)

However, in most groups, participants expressed little or no concern about secondhand harms from vaping. Isaac (17, never-smoker, never e-cigarettes), for example, stated: “I think they're safe for, like, other people around you because it's not…it doesn't give the black smoke off so…that black carbon monoxide thing. It just gives out those chemical things.”

All groups were asked what harms, if any, they considered might be associated with e-cigarettes. Nicotine dependency was briefly mentioned as a potential harmful effect of e-cigarette use in several groups. A key issue mentioned was that e-cigarettes could be used by established smokers to maintain their nicotine addiction in indoor spaces where traditional cigarettes were banned (see the Results section). A few participants drew comparisons of nicotine addiction to ‘caffeine’ addiction, and several compared nicotine harms with tobacco harms. Thomas (16, never-smoker, never e-cigarettes) postulated: “nicotine is bad for you. I know it's not as bad, I don't think it's as bad as a traditional cigarette because it doesn't have the other effects of like the blackening of your lungs and stuff. But nicotine, having too much nicotine in your blood is really bad, especially if you're having children”.

Assumptions that e-cigarette use was low risk seemed to be at least partially influenced by their marketing, appearance, packaging and the absence of health warning labels and other regulation. Janice (15, never-smoker, never e-cigarettes) reflected: “Like, cigarettes with…well, I personally associated them with things like cancer, things like death, ‘cause it says on a packet ‘Smoking kills’. But then I kind of well think that if I saw somebody smoking an e-cigarette I'd think that was beneficial and more kind of harmless […] e-cigarettes look, well, innocent.”

### Appeal of e-cigarettes to children and teenagers

E-cigarettes were described as easy to obtain, ‘fun’ products, with ‘direct appeal’ to teenagers and children due to the wide variety of ‘great flavourings’, ‘bright colours’ and ‘fun tricks’ commonly associated with them. Participants expressed particular concerns that e-cigarettes were ‘inappropriate’ for*,* yet ‘attractive to children’*.* Several potential reasons were given for their appeal to children/young teenagers. Nine groups explicitly discussed e-cigarettes as ‘a safer alternative to smoking for teenagers’ and as a less harmful means of rebelling, for example:

Darnell “For people that age [14] it [e-cigs] seems to be sort of the best of both worlds. You get to sort of rebel against the teachers ‘cause you're doing something they don't necessarily want you to do, but it doesn't necessarily come with the harm of a cigarette.” (14, never-smoker, tried e-cigarettes)

Jane “*Or as much.”* (17, tried-smoking, tried e-cigarettes)

Darnell “Or as much, so it's sort of a, a win-win.”

In another group, participants similarly discussed e-cigarettes' appeal to young teenagers, particularly those ‘scared of using proper ones’, wanting to try out the experience of ‘smoking’ without exposing themselves to the associated harms and adult opposition:

When we were younger, like, we had fags. We used to go out to a smoker corner and smoke fags. I think now in this like generation, they've got these wee e-cigarettes. And if they see them, the parents are gonna be like, oh, as long as they dinnae smoke fags I guess it's all right. (Stewart, 17, current smoker, current e-cigarettes)

‘*Fitting in’* and ‘*looking cool’* were other explanations for the appeal of e-cigarettes, illustrated by the following quote and exchange:

Some people are like, they use these ‘cause […] they still wanna fit in, so they'll turn to this. […] If a kid, a kid's trying to fit in with someone else, they're gonna look what they're doing, aren't they? If they're [others are] smoking an e, like, an electronic cigarette, they're gonna wanna do that to fit in with them, aren't they? (Finlay, 15, never-smoker, never e-cigarettes)

Rhyan “You get the younger ones who start on e-cigs, they're like smoking but they're not and they just walk around with hoods up thinking they're hard.” (17, tried-smoking, tried e-cigarettes)

Katie “*Gangsters wannabe.”* (17, tried-smoking, never e-cigarettes)

### Acknowledgement of e-cigarette experimentation, use and dual use among different user groups

In 13 groups, there was consensus that the primary beneficiaries of e-cigarettes were long-term smokers, often explicitly described as ‘old’, ‘middle-aged’ and of an older generation. E-cigarettes were frequently described as benefiting ‘smokers who wanted to quit’. Charity (16, never-smoker, never e-cigarettes) voiced concerns about whether e-cigarettes had lower capacity to satisfy cravings and reduce stress and therefore whether they could help smokers quit entirely: “smokers and, like, they go onto these [e-cigarettes], they might start realising that they're having no benefit off ‘em an’ then, like, with smoking they had more benefit, they're feeling more relaxed and less stressed, whereas with these they're just blowing in smoke and puffing it back out”.

In two groups, participants with personal experience of smoking and vaping suggested that e-cigarettes could have a role in helping children and teenagers ‘maintain’ (Lauren, 16, never smoked, never e-cigarettes) a nicotine addiction, with participants in one group noting that e-cigarettes offered young underage smokers new opportunities to ‘hide’ the fact that they were smokers, while satisfying their ‘cravings’. Louisa (16, current smoker, current e-cigarettes), for example, explained this to other group members: “I can use it [e-cigarette], like, in my house ‘cause my mum doesn't know I smoke so then I could just use that when I'm, like, feeling like I need a fag and then the cravings just disappear so it's quite good.” Similarly, Danyul (16, current smoker, current e-cigarettes) mentioned feeling “less guilty about mixing the two to up levels” (smoking and vaping to get nicotine).

Underlining their youth appeal, nine groups discussed e-cigarettes as fun products used in social contexts such as parties. Some participants considered e-cigarettes as almost exclusively ‘a party thing’, reporting themselves as unlikely to use them when alone. Fergus (17, current smoker, current e-cigarettes) explained how: “they're good because, like, at a party, if you're going out to a party, you never talked about fags, you just said can I get a fag? Like now people are saying, ‘have you tried this flavour, have you tried this colour?’ […] it's a conversation-starter.” In one group, high nicotine content e-cigarettes were deemed best because: “it makes like young people high as well ‘cause, like, it gets, like, it's like the same feeling as getting drunk. A nicotine buzz” (Matthew 17, current smoker, tried e-cigarettes). However, some data suggested their appeal levelled off after a while, with David (16, tried-smoking, tried e-cigarettes) reporting that e-cigarettes: “were kind of popular in my school, like about a year ago like we'd bring them to parties. […] at that point it was seen as cool but now it's sort of seen as a joke. Everyone seems to think they're like kinda, they're a bit embarrassing really”.

Building on discussions about their youth appeal, the groups were asked to reflect on whether e-cigarettes encouraged children and young teenagers to try traditional cigarettes, with seven groups discussing this as a possibility. Thus, Rhyan (17, tried-smoking, tried e-cigarettes) stated: “I think if you're not noticing the whole benefit of e-cigarettes then you probably will start going onto the harsher things. It's like the same issue with drugs—like, once you've got addicted to that kind of drug and then you realise that it's not giving you the type of high you're looking for, you start going onto the harder substance.” Most of these discussions were speculative rather than based on experience. Only one participant, Finlay (15, never-smoker, never e-cigarettes), mentioned knowing people who had first used e-cigarettes; he drew on observations of peers who he recalled experimenting with e-cigarettes before moving to traditional cigarettes. He said: “I know a couple of people, of, like, they haven't smoked before but they've tried these on, like, a one-off basis, and then tried them again and started using them regularly, and then just moved onto, like, proper tobacco smoking and that.” Contemplating whether e-cigarettes were considered an adequate substitute for and authentic alternative to, traditional cigarettes, Alex (17, current smoker, tried e-cigarettes) concluded switching would be possible either way: “smokers go from the normal cigarette to that [e-cigarette] because they're still getting the sensation of smoking a cigarette. So it would be quite easy to go the other way.” Opposing such views, Lesley (17, tried-smoking, never e-cigarettes) firmly rejected the idea of e-cigarettes as a gateway, stating: “Naw, I don't think […] if you started smoking e-cigarettes in the first place it'd be a bit dumb to go onto smoking cigarettes”.

### Interplay between online and real-world experiences

Participants identified smokers as the primary target audiences for e-cigarette marketing, but acknowledged that, because of their appeal to young people and non-smokers, this ‘intended’ target group might be expanding. An issue which emerged spontaneously in six groups was the appeal of online YouTube videos showing vaping tricks, with conversations becoming good humoured as participants eagerly described particular tricks. Participants referred to the pervasiveness of these videos. For example, Caiomhe (15, never-smoker, never e-cigarettes), an avid Facebook user, said that vaping trick videos were “all I saw [on Facebook] for like, for like three weeks, that was like literally everything like I saw.” Jonathan (17, never-smoker, never e-cigarettes) noted how: “The reach of those videos via social media's absolutely incredible. […] you see people's accounts and you're like, ‘Who on earth are they that they come from like the other side of the planet, they would have no idea I even exist, yet I'm watching their video.’” Vaping tricks videos seemed to encourage imitation: Gregor (17, current smoker, current e-cigarettes) had tried “to make a hula hoop [using an e-cigarette] and failed.” There was general ambiguity about who might be responsible for posting and promoting such videos on social media sites. Their viral nature and popularity, however, were frequently highlighted, with participants confidently stating: “You always see these cool videos of people doing them smoke tricks and I think that's how e-cigs became so popular” (Charity, 16, never-smoker, never e-cigarettes).

## Discussion

This focus group study provides one of the first rich descriptions of teenagers' lived experiences and perceptions of e-cigarettes. They generally viewed e-cigarettes as useful products for smokers, to quit or reduce traditional cigarette use. Although framed within considerable uncertainty, participants viewed e-cigarettes as less harmful than traditional cigarettes, believing the health risks from secondhand vapour to be lower than from traditional smoking which is consistent with current evidence.^28^ E-cigarettes were considered appealing to some participants since they were available in fun flavours and provided a relatively safe way to rebel. While all e-cigarette users were also smokers in our sample, teenagers' use of e-cigarettes appears to be strongly influenced by their social environments. This resonates with Corsi and Lippert's^29^ recent work which examines the use of e-cigarettes in US schools and suggests that some school environments facilitate e-cigarettes use. They described using e-cigarettes in a variety of situations, including at parties or, for a few smokers, when they felt they could not smoke traditional cigarettes. All of the teenagers who had used e-cigarettes had purchased them over the internet or been given them by other teenagers.

Our study adds to existing concerns about teenagers' perceptions of e-cigarettes as fun, cool products,^30^ ^31^ by considering the role of marketing via (social media) channels frequently accessed by, and appealing, to this age-group. Previous research has documented the magnitude of exposure to e-cigarettes (as well as alcohol and tobacco) that occurs when viewing YouTube videos,^32^ and has also shown that portrayals of smoking in popular films are associated with uptake of smoking in adolescents. Our findings add to this by demonstrating the potential mechanisms by which exposure to e-cigarette materials online may influence attitudes and resultant behaviours. While restrictions on mass media marketing of e-cigarettes are increasingly considered internationally, social media and online environments are more difficult to manage because user-generated content (in particular) will not be covered by incoming regulations. Our study findings resonate with a larger US study which found that teenagers were easily able to purchase e-cigarettes from the internet because of an absence of age-verification measures used by internet vendors.^33^

A key issue of focus for policy has been the potential for a ‘gateway’ effect to be operating. While this term has been used in varying ways, its key feature is the idea that consumption of a less risky product (such as e-cigarettes) leads to the use of a more harmful product.^34^ At present, there is a lack of consensus as to whether a gateway effect operates or how it could be measured. Two recent US-based longitudinal studies have suggested some adolescents may have used e-cigarettes prior to cigarette smoking,^15^ ^16^ but other research has found no evidence of increased cigarette smoking.^35^ ^36^ It is noteworthy that all current e-cigarette users were dual users, and only one participant reported indirect evidence of e-cigarette users going on to become regular cigarette smokers. However, our study raises the possibility that an alternative effect may operate: a few of these teenagers who used e-cigarettes discussed the opportunities e-cigarettes offer to them to covertly vape (hiding nicotine intake from parents/others). In some teenagers, this may reinforce nicotine use at a life stage when experimentation with tobacco smoking is common, which might foster the transition to regular smoking. Teenage e-cigarette users have previously noted the covert nature of e-cigarettes as a perceived advantage in a small US-based qualitative study^37^ ^38^ and in a larger questionnaire US study, authors conclude that e-cigarettes may contribute towards subsequent cigarette use via nicotine addiction or social normalisation of smoking behaviours.

## Conclusions

Teenagers support e-cigarettes as smoking cessation aids for established adult smokers. They engage with these products in a different manner to traditional cigarettes. E-cigarettes are perceived as attractive to some teenagers and as offering a new product for experimentation, encouraged via online videos and social media. While we find little direct evidence of a ‘gateway effect’ among our participants, we identify a novel hypothesis worthy of further investigation: e-cigarettes' potential for covert use may reinforce traditional cigarette smoking in teenagers. These findings suggest policy responses should more clearly meet the needs of young people, as well as helping established adult smokers.
